# Provincial alcohol index and its relationship to alcohol-related harm in Thailand: implications for subnational alcohol policy development

**DOI:** 10.1186/s12889-016-3217-4

**Published:** 2016-07-11

**Authors:** Surasak Chaiyasong, Thaksaphon Thamarangsi

**Affiliations:** Social Pharmacy Research Unit (SPRU), Faculty of Pharmacy, Mahasarakham University, Kantharawichai, Maha Sarakham 44150 Thailand; Health Promotion Policy Research Center (HPR), International Health Policy Program (IHPP), Ministry of Public Health, Muang, Nonthaburi 11000 Thailand

**Keywords:** Alcohol consumption, Alcohol-related harm, Thailand, Alcohol policy

## Abstract

**Background:**

The Provincial Alcohol Index (PAI) is one of the efforts to develop a composite measurement to operationalize the situation of alcohol consumption and related risk behaviors. The index offers a means for national and subnational alcohol control committees to address alcohol-related problems in their responsible jurisdiction areas. The objective of this study is to assess the relationship between PAI scores and alcohol-related problems using Thailand as an example.

**Methods:**

Cross-sectional analyses of PAI scores based on the 2007 National Cigarette Smoking and Alcohol Drinking Behavior Survey (CSAD) and the National Statistical Office data were conducted. CSAD data were collected from 168,285 Thai residents aged 15 years and above in 76 provinces of Thailand (population range 180,787 to 5,716,248). The PAI scores were generated using three different methods based on five indicators: 1) prevalence of adult (≥15 years) drinkers, 2) prevalence of underage drinkers, 3) proportion of regular drinkers, 4) proportion of binge drinkers and 5) proportion of drink-drivers. Alcohol-related injuries and violent events together with provincial level covariates (age, gender, income and region) were assessed. Correlational and linear regression analyses were performed to examine the relationship between PAI scores and alcohol-related problems.

**Results:**

The PAI scores generated from the three methods were significantly correlated with one another (*r* > 0.7, *p* < 0.05) and significantly related to alcohol-related problems after adjusting for the provincial level covariates. Based on the normalized method, PAI scores had a significant and positive relationship with prevalence of alcohol-related injuries (beta = 562 cases per million population, *p* = 0.027) and violence (beta = 451 events per million population, *p* = 0.013). PAI scores were highest in the north and lowest in the south of the country.

**Conclusions:**

The findings of this study illustrate the relationship between the PAI and alcohol-related problems. The PAI scores can be used to benchmark the alcohol situation across jurisdiction areas. Future studies are suggested to develop a scale to measure subnational alcohol policy performances.

## Background

Alcohol is the leading health risk factor in Thailand, attributable to up to 10 % of the total burden of disease in terms of disability adjusted life years [1]. Compared to the global figure of 5.1 % [2], the alcohol-attributable health burden in Thailand is almost two-fold higher. Thailand enacted the Alcoholic Beverage Control Act in February 2008, the first major law to prevent and control alcohol related burden of disease. This Act has both a regulation section and a system and process section. In the regulation section, the Act focuses on alcohol availability including condition and place of sales, purchasers, retail sellers, drinking venues and alcohol promotion and marketing. In the system section, the Act establishes Alcohol Control Committees at both the national and provincial levels [3].

In 2009, the second National Health Assembly adopted the National Alcohol Policy Strategy, designed to promote a concerted effort to guide alcohol policies and actions at both the national and local levels. The Strategy recommends five major strategies and 10 general and intervention-specific indicators to monitor and evaluate the progress. The five strategies include controlling price and availability, modifying attitudes (marketing control, education and persuasion), risk reduction, alcohol policy in every setting, and supportive mechanisms for alcohol policy [4]. The indicators include alcohol consumption (e.g., prevalence among adult (aged 15 years and above) drinkers and underage (under 20 years) drinkers, quantity of drinking per occasion), drinking patterns (e.g., percentage of regular and binge drinkers), alcohol-related behaviors (e.g., percentage of drink-drivers) and negative consequences (e.g., rate of alcohol dependence, alcohol use disorders, and alcohol-related injuries) [4].

Although Thailand has had both a national strategy and legal tool to control alcohol consumption for many years, consumption and related problems among groups who are traditionally abstainers has not decreased [3]. Weaknesses and obsoleteness of alcohol policy content and poor implementation, together with the increase in alcohol marketing, are common explanations. Therefore, it is crucial to continue and strengthen efforts to address alcohol problems in Thai society, particularly at the local level.

Evidence is essential for guiding alcohol policy, and can be used to promote competition across jurisdiction areas and among alcohol control partners. Developing feasible, understandable and meaningful parameters to monitor the situation, however, is not straightforward. At the global level, the World Health Organization (WHO) has developed and reported many indicators to promote efforts to address alcohol problems, such as adult per capita consumption (APC), prevalence of drinking and rate of heavy episodic drinking [5], score of harmful drinking pattern [6] and price of alcoholic beverages adjusted by living cost [6]. A recent study recommends two sets of minimal indicators: APC, prevalence of abstention, and frequency of binge drinking to monitor alcohol exposure, and alcohol-attributable years of life lost due to premature death to examine alcohol-related health consequences [7]. In Thailand, APC, drinker prevalence and road traffic mortality rate are common indicators of alcohol consumption.

Composite index is another way to accommodate multiple indicators and is not new to the alcohol policy arena. Several studies have introduced a composite index to measure and compare situations on alcohol consumption, drinking patterns, drinking determinants and strength of alcohol policies and its implementation [8–13]. Relevance, accuracy, consistency and applicability of these indices across different contexts are still a concern. Theoretically, a composite index should measure multidimensional concepts which cannot be captured by a single indicator. In addition, the index should be linked to other measures to test its explanatory power [14].

For alcohol policy, Brand et al. developed a composite index of policy strength that accounted for relative policy efficacy and policy implementation for 30 countries [9]. Their index score has a strong relationship with per capita consumption [9] and youth drinking [15]. Using aggregate and individual-level data from 15 countries, Cook et al. found that regulating physical availability of alcohol was associated with lower alcohol consumption in low- and middle-income countries [13]. Recently, Naimi and colleagues developed a scale to measure the aggregated state-level alcohol policy environment and to assess its association with state-level adult binge drinking prevalence in the United States [12]. They generated a score based on 29 policies by five different methods and found that the scores were significantly correlated with each other and all scores were significantly associated with adult binge drinking prevalence.

For alcohol consumption, Gmel et al. developed a scale to measure the level of detrimental drinking patterns which were used for the Comparative Risk Analysis in the 2000 Global Burden of Disease study [10]. This scale comprises four aspects of drinking patterns, including quantity of consumption per drinking day, frequency of drinking, risky single-occasion drinking, drinking with meals and drinking in public places. Based on survey data from the GENECIS project, this scale was validated in various countries and the scale score had a positive association with alcohol use disorder symptoms and alcohol-related injuries and physical altercations at individual and aggregate levels [8].

There have been efforts to develop composite indices on alcohol and later promote their use in Thai national and local alcohol policy processes. This includes comparison of alcohol-related situations across provinces [3, 16, 17], with the primary aim to enhance the proactive role of Provincial Alcohol Control Committees.

The Cigarette Smoking and Alcohol Drinking Behavior Survey (CSAD) is a national survey on tobacco and alcohol use conducted by the National Statistical Office (NSO) every 3 years. Recent CSAD surveys cover both alcohol consumption, pattern of use, drinking determinants and a few selected harmful outcomes. Although it has been conducted for more than two decades, it was not until 2007 that the survey represented every province in Thailand [18]. Thus, the 2007 CSAD is the first opportunity for full provincial comparison, where alcohol control committees and policy-makers can monitor the situation of alcohol consumption and consequences in all provinces over time.

The Provincial Alcohol Reports [16, 17] introduced the Provincial Alcohol Index (PAI) to compare various alcohol indicators across provinces. These composite indices were developed from available data obtained from the surveys. In the Report, the 2007 PAI covers three indicators: drinker prevalence, percentage of regular drinkers and percentage of binge drinkers, while the 2010 PAI included two more indicators: prevalence of underage drinkers and percentage of drink-drivers to cover additional dimensions of alcohol-related risk behaviors. The Provincial Alcohol Report has gained attention from public media as well as policy makers, particularly in high-risk provinces, and has led to numerous initiations of prevention and intervention campaigns at the local level [19]. However, the extent to which the PAI accounts for different alcohol-related problems is still unknown. The objective of this study was to assess the relationship between PAI scores and alcohol-related problems in Thailand. It will help shed light on a tool to move subnational alcohol policy, using Thailand as a good example.

## Methods

This study obtained data on alcohol consumption and alcohol-related harm from the CSAD in 2007. PAI scores were generated by three different methods. Correlation of the scores obtained from the different methods was examined and presented descriptively. The relationship between PAI scores and alcohol-related problems was assessed using multiple linear regression models.

### Data sources

Data on alcohol consumption and related behaviors were obtained from the 2007 CSAD. The CSAD is a two-stage stratified survey, administered by the NSO every 3 years. The survey sampling design and methodology is described in detail elsewhere [20]. The 2007 CSAD collected data from residents in all provinces (population range 180,787 to 5,716,248) with a final sample size of 168,285 respondents aged 15 years and above. The response rate was 83.93 %. The sample was representative of the national population and provincial populations. Respondents were assured of their anonymity and confidentiality of their responses, that there were no right- or wrong-answers, and were told to answer the questions as honestly as possible. Together with the fact-based questionnaire items, alcohol indicators and alcohol-related harms estimated from the same survey were unlikely to be associated with common method variance [21]. The key variables of alcohol consumption and related behaviors together with sampling weights, the estimates that reflect the probability of respondent selection, were used to generate the PAI scores and alcohol-related problems estimates at the provincial level. Socio-demographic data, including total population, proportion of males and females, age differences in the population, and per capita income were obtained from the NSO.

### Provincial alcohol indicators

We used three different methods to calculate the PAI. Principles for the PAI development include being meaningful, traceable over time and user-friendly. With regard to recommended indicators and identified high-risk drinking and related behaviors in the National Alcohol Policy Strategy and Provincial Alcohol Reports, together with availability of data at the provincial level, five indicators were selected to develop the PAI. These indicators are: prevalence of adult drinkers, prevalence of underage drinkers, proportion of regular drinkers, proportion of binge drinkers and proportion of drink-drivers. Based on CSAD, drinkers were defined as persons who consumed alcohol in the past 12 months; this was drawn from the question “Had you drunk any alcoholic beverage in the past 12 months?” Regular drinkers were defined as drinkers who consumed alcohol at least once a week, based on the question “How often did you drink alcohol in the last 12 months?” Binge drinkers were defined as drinkers who consumed 5 or more standard alcoholic beverages (50 g or more of ethanol) per drinking occasion; the question was “How often did you heavily drink alcohol per drinking occasion in the past 12 months?” with definition of heavy drinking per occasion as five or more drinks of spirits, beer, wine or ready-to-drink. Drink-drivers were defined as persons who drove any auto vehicles after drinking, derived from the question “Had you driven any auto vehicles after drinking alcoholic beverage in the past 12 months?” The denominator for the last three indicators was the total number of drinkers in the past 12 months prior to the survey. All indicator scores were estimated using the sampling weight to account for a complex survey design.

Method 1 is a modification of the drinking pattern score for the Comparative Risk Analysis [10]. This ‘criteria-based method’ uses the sum of the five dichotomous scores, 0 or 1, for each of the five indicators. The PAI scores from this method hence range from 0 to 5. In calculation of the drinking pattern score, a previous study used 0.5 as the cut-point for prevalence-based indicators and 312/365 days as the criteria for frequent drinkers [10]. As Thailand is a low-prevalence country in terms of alcohol consumption, we used the national averages for all cut-points (0.299 for adult drinkers, 0.127 for underage drinkers, 0.558 for regular drinkers, 0.164 for binge drinkers and 0.347 for drink-drivers). Method 2 used an aggregate of the actual proportion of all indicators (range 0 to 1), rather than dichotomized estimates used in Method 1. Method 3 normalized each indicator before aggregating the scores. This procedure involves transforming the indicators so that they have an identical range (0 to 1) by subtracting the minimum value and dividing by the range of the indicator values. The scores for each province and each of the five indicators were constructed using the following formula:

$$ {\mathrm{S}}_{\mathrm{pi}}=\left({\mathrm{I}}_{\mathrm{pi}}\hbox{--} { \min}_{\mathrm{i}}\right)/\left({ \max}_{\mathrm{i}}\hbox{--} { \min}_{\mathrm{i}}\right) $$

where ‘i’ represents the indicator (i =1, …, 5) and ‘p’ the province (*p* = 1, …, 76); S_pi_ = scores for province ‘p’ in relation to the indicator ‘i’; I_pi_ = value for indicator ‘i’ in province ‘p’; min_i_ = minimum value of indicator ‘i’ among the provinces; max_i_ = maximum value of indicator ‘i’ among the provinces.

In each province, the PAI scores were then averaged over the five indicators:

${PAI}_p=\sum_i{S}_{pi}/n$, where ‘n’ is the number of indicators used to generate the PAI scores.

For all methods, the PAI scores were scaled up to 100 to improve clarity. Table 1 shows an example of the three methods to calculate the PAI scores for Bangkok.

**Table 1:** National estimates and a provincial example of five indicators and PAI scores

	National estimates			Bangkok’s estimates			
	Average	Minimum	Maximum	Actual value	Method 1	Method 2	Method 3
Indicator							
Prevalence of adult drinkers	0.299	0.022	0.545	0.212	0	0.212	0.364
Prevalence of underage drinkers	0.127	0.070	0.332	0.074	0	0.074	0.224
Proportion of regular drinkers	0.558	0.363	0.727	0.572	1	0.572	0.575
Proportion of binge drinkers	0.164	0.030	0.353	0.184	1	0.184	0.477
Proportion of drink-drivers	0.347	0.121	0.629	0.121	0	0.121	0
PAI score					40.0	23.3	32.8

Note: The PAI scores were scaled up from 1 to 100. For method 3 calculation using Bangkok as an example, score for the indicator of adult drinker prevalence is that (Bangkok actual value – minimum value)/(maximum value – minimum value) = (0.212–0.022)/(0.545–0.022) = 0.363. (The numbers in Table 1 may be different from calculation due to rounding methods.)

### Alcohol-related harms

With regard to availability of alcohol-related harm data at the provincial level, two groups of alcohol-related harms: alcohol-related injuries and alcohol-related violence were obtained from the 2007 CSAD. In the survey, these two acute harms covered alcohol-related problems caused by respondent’s drinking and other’s drinking and were collected from all respondents (drinkers and nondrinkers). Alcohol-related injury was a self-report of experiencing any injuries or accidents related to alcohol consumption. Alcohol-related violence, also self-reported, was defined as an experience of violence or assaults due to alcohol consumption.

### Analysis

This study employed Pearson’s correlation coefficient to determine pairwise correlations between the PAI scores for all 76 provinces developed by the three methods. Linear regression was used to examine the relationship between PAI scores and alcohol-related harms. Goodness of fit for each model was evaluated using the R-squared. Multiple linear regression models were used to compare differences in PAI scores across provinces with adjustment for socio-demographic variables. A map of Thailand taken from the Burden of Disease Thailand, International Health Policy Program (see http://thaibod.net/webapp/BOD/) was used to show variations in provincial PAI scores.

## Results

### Provincial alcohol index scores

The PAI scores ranged from 0 to 100 for method 1, 17.42 to 39.82 for method 2 and 17.46 to 69.32 for method 3. The prevalence of alcohol consumption and alcohol-related risk behaviors differed widely across provinces (data not shown). The distribution of PAI scores from all three methods was normal. The northern provinces had higher scores compared to the southern provinces. Based on all the three methods, Phrae, a province in the upper north, had the highest score whereas Yala, a Muslim-dominated province in the southern region, had the lowest score.

### Correlation between methods

The PAI scores calculated from the three methods were all significantly correlated with one another (*r* > 0.7, *p* < 0.05). Scores calculated from methods 2 and 3 had the strongest correlation (*r* = 0.990, *p* < 0.001).

### Relationship between PAI scores and alcohol-related problems

This study found that PAI scores calculated from all three methods had a statistically significant relationship with prevalence of alcohol-related injuries. A significant relationship was also found between all PAI scores and prevalence of alcohol-related violence (Table 2).

**Table 2:** Relationship between Provincial Alcohol Index scores and alcohol-related problems

PAI score method	Beta	SE	*p*-value	R-squared
Alcohol-related injuries				
Method 1	284.67	83.69	0.001	0.135
Method 2	1442.13	453.57	0.002	0.120
Method 3	614.29	194.36	0.002	0.119
Alcohol-related violence				
Method 1	149.09	59.06	0.014	0.079
Method 2	835.81	316.12	0.010	0.086
Method 3	368.05	135.00	0.008	0.091

*SE* standard error

Table 3 shows results of the multiple linear regression model predicting the number of alcohol-related injuries using method 3, the method used in Thailand Provincial Alcohol Report, to calculate PAI scores. After including provincial level covariates such as percentage of residents aged 20 years and above, percentage of males, income per capita and region, the goodness of fit of the model slightly increased. The coefficient for PAI score was 562, indicating that for every unit increase in PAI score, the prevalence of alcohol-related injuries would increase by 562 cases per million population (*p* = 0.027). In addition, the PAI score was also significantly associated with alcohol-related violence (beta = 451 events per million population, *p* = 0.013).

**Table 3:** Relationship between alcohol-related injuries and alcohol-related violence and provincial level variables from the multiple linear regression model

Provincial level variable	Beta	SE	*p*-value
Alcohol-related injuries			
PAI score	562.06	249.39	0.027
Percentage of males	3567.72	3474.89	0.308
Percentage of adults aged >20 years	−578.79	1072.41	0.591
Income per capita (THB1000)	−3.38	17.02	0.843
Region			
Bangkok (reference)	–		
Central	−336.65	19,719.45	0.986
Northern	−8030.17	21,137.76	0.705
Northeastern	−9126.52	21,324.11	0.670
Southern	−14,855.61	21,227.79	0.486
Alcohol-related violence			
PAI score	450.52	176.12	0.013
Percentage of males	1136.49	2453.92	0.645
Percentage of adults aged >20 years	89.65	757.32	0.906
Income per capita (THB1000)	−3.67	12.02	0.761
Region			
Bangkok (reference)	–		
Central	2400	13,925.60	0.864
Northern	−2881.27	14,927.19	0.848
Northeastern	−3489.48	15,058.79	0.817
Southern	3072.55	14,990.77	0.838

Note: PAI scores were calculated using the normalized method. The outcomes were prevalence of alcohol-related injuries per 1 million population and prevalence of alcohol-related violence per 1 million population

*SE* standard error

**Fig. 1:**
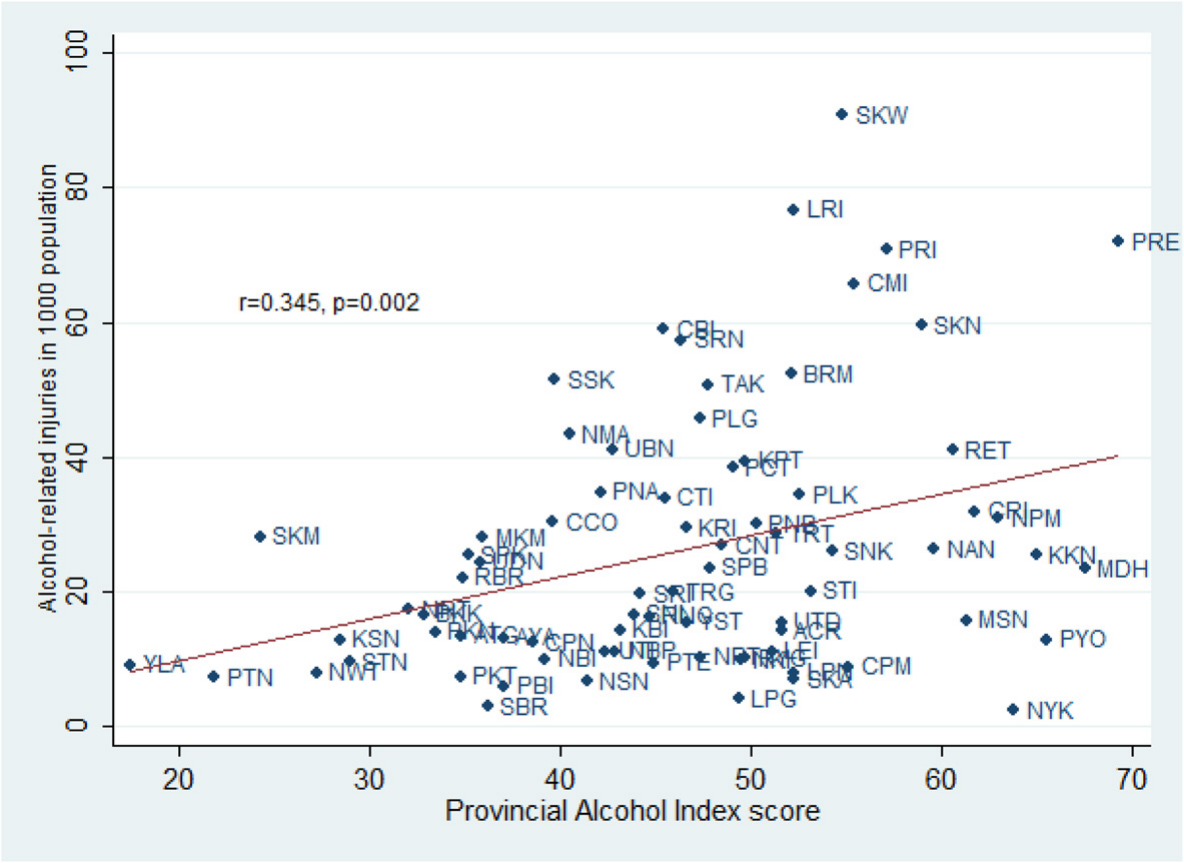
Scatterplot of Provincial Alcohol Index scores and alcohol-related injuries for each province. The scores were estimated using the normalized method

Figure 1 shows the relationship between provincial PAI scores using method 3 with prevalence of alcohol-related problems. The five provinces with the highest PAI scores were Phrae, Mukdahan, Khon Kean Phayao and Nakhon Nayok. Of these, Phrae, Mukdahan and Khon Kean had a prevalence of alcohol-related injuries above the national median (20,119 cases per one million population). The five provinces with the lowest PAI scores were Yala, Pattani, Narathiwat, Kalasin and Samut Songkhram. Of these, all except Samut Songkhram had a prevalence of alcohol-related injuries below the national median.

**Fig. 2:**
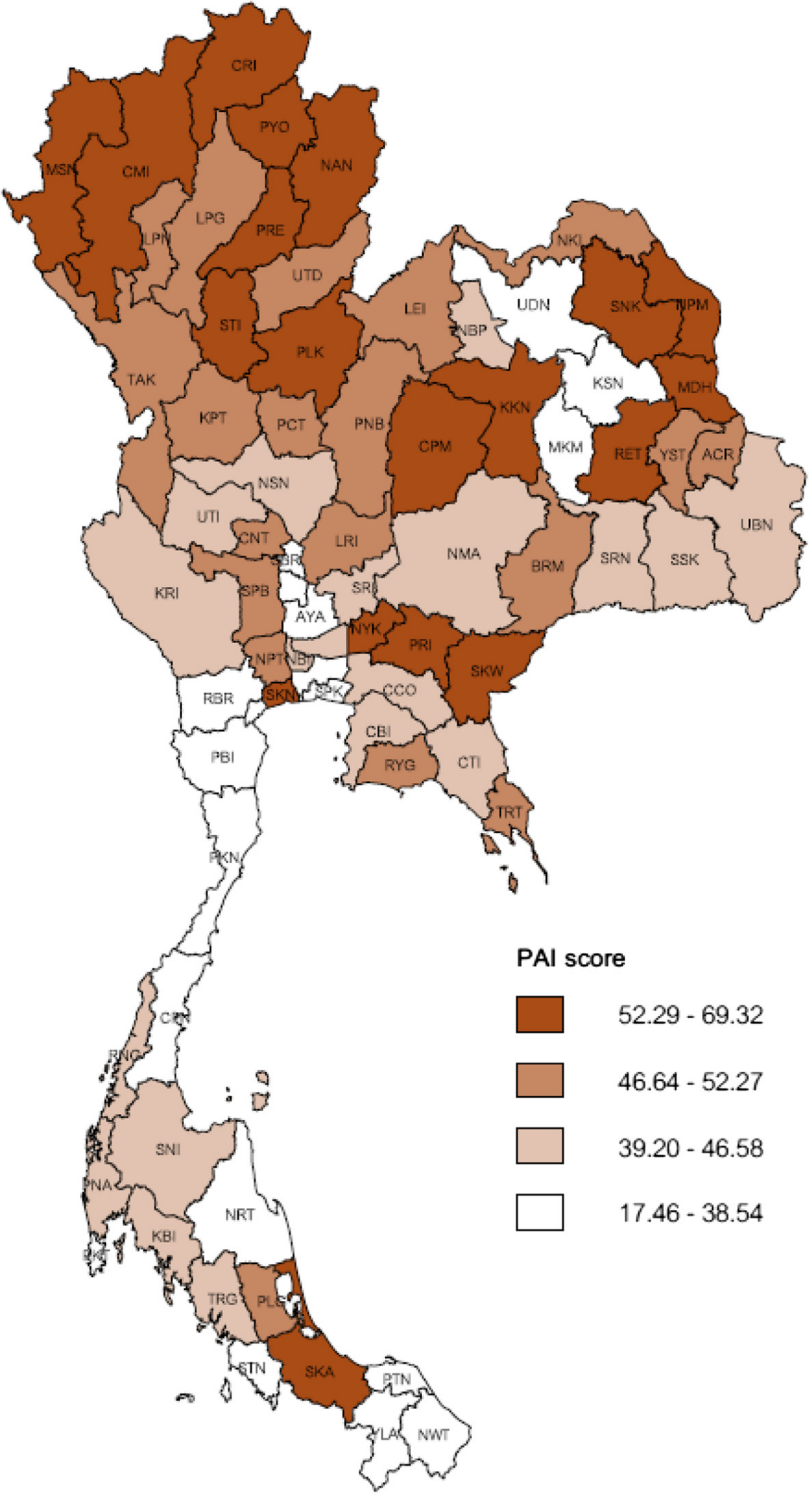
Map of Thailand with the Provincial Alcohol Index scores. The score was estimated using the normalized method. A map of Thailand was taken from the Burden of Disease Thailand, International Health Policy Program (http://thaibod.net/webapp/BOD/)

Figure 2 displays a thematic map of Thailand showing variations in provincial PAI scores. The darkest shade represents the highest quartile while the lightest shade represents the lowest quartile. All provinces in the northern region tended to have higher PAI scores, while the southern provinces tended to have the lowest scores, an exception being Songkhla. The central and north-eastern regions had wide variations in PAI scores.

## Discussions

This study found that PAI scores were significantly and independently associated with alcohol-related problems. The study findings confirm results of previous studies examining associations of detrimental drinking patterns with alcohol-related problems [8, 22, 23]. To our knowledge, the Provincial Alcohol Index is one of few efforts to develop and validate a composite measurement to operationalize the situation of alcohol consumption and related risk behaviors in Thailand. This study is important to determine the extent to which the alcohol consumption situation measure accounts for differences in alcohol-related problems across provinces in Thailand and inform local policy makers.

Findings were similar regardless of the method for calculating the PAI scores. This indicates that the approach was robust with respect to operationalizing the alcohol consumption situation across several methodologies [12]. Methods 2 and 3 had the highest correlation, most likely because they generated the scores using a very similar approach. Using the actual values of each component to operationalize the PAI is a more appropriate method than the criteria-based method. The main difference between Method 2 and 3 concerns the range of scores; method 2 aggregated the proportions of drinkers and high-risk drinkers and subsequent behaviors provided a narrow range of scores (from 17.4 to 39.4). Normalizing these proportions across provinces, as was done in Method 3, extended the range of the scores to between 17.5 and 69.3. The narrower range from Method 2 may reflect drinking norms in Thai society with low prevalence of drinkers and detrimental drinking behaviors [24]. Method 2 is simple and easy for provincial authorities to compare PAI scores with other provinces. However, for comparing PAI scores over time, changes in scores may be biased due to the so called “regression toward the mean” effect. Hence, we recommend method 3, which normalizes the scores and is more appropriate to use when comparing the scores over time.

The PAI score is comprised of five indicators which were treated equally. Some studies applied weights to the indicators before aggregation [9, 12]. However, the indices used in these studies were alcohol policy scales, where valid reasons for applying the weights could be made by measuring the impacts of different alcohol policy interventions. To our knowledge, researchers have not recognized any weighting approach applied for alcohol consumption indicators. It may be true that different consumption patterns may have a different extent of effects. However, they all are detrimental and the variation may not be consistent across jurisdictions and cultures. For example, it may not be valid to say binge drinking is more harmful than regular drinking in all settings. Therefore, we argue for a non-weighting approach for calculation of PAI scores, at least until we have a better understanding on the different impact of various harmful drinking patterns in Thai society.

This study found that PAI scores had a significant positive relationship with prevalence of alcohol-related injuries and alcohol-related violence, although the goodness of fit of the model was low. One explanation for this finding is that the PAI score does not include quantity of drinking per occasion which has a strong relationship with alcohol-related problems [2, 25].

Alcohol consumption and related risk behaviors differed widely across provinces of Thailand. The prevalence of drinking was highest in the north and lowest in south. This is partly due to higher availability of alcohol as well as cultural differences in drinking. Production of both industrial and locally made alcohol is higher in the north. Alcohol is part of many events and activities in the north, while its use is prohibited among the majority Muslim population in the south [2, 3, 24]. However, among drinkers, the proportion of regular drinkers is actually higher in the south compared with the north.

The PAI offers a means for national and provincial alcohol control committees to address alcohol-related problems in their jurisdictions and encourage competition to reduce the alcohol-related burden. Furthermore, the PAI can be used to monitor alcohol consumption and harms over time as well as for assessing outcomes of the province performance in reducing alcohol consumption and its consequences. One possibility for these provincial bodies is to strengthen alcohol policy implementation and law enforcement, including development of local surveillance mechanisms for violation of regulations. These demands are very relevant to the Alcoholic Beverage Control Act and the National Alcohol Policy Strategy [4]. To date, the Provincial Alcohol Index has been used by the Thai Health Promotion Foundation and the Parliament to prioritize provinces with high risk alcohol-related outcomes (those with high index scores). In 2013, provinces in the northern region were urged to reduce their alcohol-related disease burden. They subsequently developed their own provincial alcohol control strategy to reduce alcohol consumption and related harm [19].

This study has limitations which should be acknowledged. The indicators of alcohol consumption and related risk behaviors used to generate the index scores were based on availability of existing data. Regarding the National Alcohol Policy Strategy and previous studies on harmful drinking pattern score [4, 10], one critical indicator was not included in this analysis, namely the quantity of drinking per occasion. This is due to limitation of the survey data used in this study [18, 20]. This study did not compose the index based on factor analysis or principle component analysis results, but rather it was based on the five aspects to monitor and evaluate situation of alcohol drinking and related risky behaviors, identified by the national strategy and provincial alcohol reports. Thus, each of those five measures was not considered to solely compose the index. The purpose of this study was to determine whether the Provincial Alcohol Index is associated with alcohol-related injuries and violence. These acute outcomes may have been underestimated since they were obtained from self-reports. However, the measurement of these outcomes should be consistent across provinces [20]. The present study did not examine the relationship of the index with chronic conditions and diseases. Although a previous study recommended years of life lost due to premature death as a minimal set of harm indicators [7], outcome data is limited by the time-lag between alcohol exposure and related chronic harm.

## Conclusions

This study showed that the Provincial Alcohol Index is a useful tool to benchmark alcohol consumption situations and consequences at the subnational level. This study also found that the composite index had a significant relationship with alcohol-related harms in Thai society. To further enhance alcohol control at the subnational level, future studies should develop an index to measure the strength of alcohol policies as well as the performance of subnational areas to prevent and control alcohol consumption and related harms.

## Abbreviations

APC, alcohol per capita consumption; CSAD, cigarette smoking and alcohol drinking survey; NSO, National Statistical Office; PAI, provincial alcohol index; WHO, World Health Organization.
